# Personality Factors and Subjective Cognitive Decline: The FACEHBI Cohort

**DOI:** 10.1155/2020/5232184

**Published:** 2020-02-19

**Authors:** Nathalia Muñoz, Montserrat Gomà-i-Freixanet, Sergi Valero, Octavio Rodríguez-Gómez, Angela Sanabria, Alba Pérez-Cordón, Isabel Hernández, Marta Marquié, Iolao Mir, Elvira Martín, Alba Benaque, Agustín Ruiz, Lluís Tarraga, Mercè Boada, Montserrat Alegret, on behalf of the FACEHBI study

**Affiliations:** ^1^Research Center and Memory Clinic, Fundació ACE, Institut Català de Neurociències Aplicades, Universitat Internacional de Catalunya, Barcelona, Spain; ^2^Department of Clinical and Health Psychology, Universitat Autònoma de Barcelona, 08193 Barcelona, Catalonia, Spain; ^3^Networking Research Center on Neurodegenerative Diseases (CIBERNED), Instituto de Salud Carlos III, 28029 Madrid, Spain

## Abstract

Individuals with subjective cognitive decline (SCD) have the perception of memory problems without showing impairment on standardized cognitive tests. SCD has been associated with an increased risk of developing Alzheimer's disease (AD). Neuroticism and openness personality dimensions have also been associated with SCD and AD. From the aforementioned, we aimed to ascertain whether the dimensions and traits defined by the Zuckerman-Kuhlman Personality Questionnaire (ZKPQ) differentiate between individuals with SCD and the general population (GP). A total of 187 participants with SCD and mild affective symptomatology recruited from the Fundació ACE Health Brain Initiative (FACEHBI) project completed the ZKPQ. Each SCD participant was matched by sex and age to an individual from the GP. Both samples included 71 men and 116 women with a mean age of 65.9 years. Results indicated that the SCD group scored significantly lower in Neuroticism-Anxiety and Activity than the GP group. Only Activity remained statistically significant in a multivariate analysis. These findings suggest that individuals with SCD have a low energy level and a dislike for an active and busy life. From the obtained results and knowing additional physical activities may delay the conversion from normal aging to cognitive impairment, we encourage promoting this lifestyle in daily routine. The assessment of personality may result in an SCD *plus* feature, which may serve as an upgrading strategy for future research.

## 1. Introduction

Subjective cognitive decline (SCD) refers to the perception of suffering from memory or other cognitive problems without objective impairment on standardized cognitive tests [1, 2]. Longitudinal studies have shown that elderly individuals referring SCD have an increased risk of progression to cognitive impairment and display functional deficits and higher prevalence of postmortem Alzheimer's disease (AD) pathology compared to those who do not perceive cognitive problems [3–8]. Determinants of SCD in aging include not only genetic and environmental factors but also individual differences in personality [3, 5, 7] that could influence the vulnerability of cognitive decline [9]. Increasing the knowledge of these factors might enhance the detection of early signs of cognitive decline and further research especially in cases with Mild Cognitive Impairment (MCI) or AD development.

Specific personality factors could be an early sign of cognitive decline. A meta-analysis with 5,054 cognitively healthy individuals showed that those with scores in the top quartile of neuroticism or in the lowest quartile of conscientiousness had a threefold increased risk of developing AD [10]. Moreover, a recent study has also reported an association between SCD and brain amyloid-*β* burden specifically among individuals with high, but not low, neuroticism [11]. Cross-sectional studies have also found SCD to be associated with AD biomarkers [1, 12–14] and with affective symptomatology, such as depression and anxiety [12, 15]. Consequently, the cooccurrence of preclinical AD signs and affective symptomatology may affect the scores obtained from personality questionnaires, but we do not know yet how they interact. Thus, studies on personality and SCD ought to control for depressive and anxiety symptoms [1, 15]. Nonetheless, most of the previously mentioned studies have only excluded participants with severe psychiatric symptomatology, i.e., displaying active major depression or been hospitalized for depression within the last year [11, 16, 17], but not those with affective symptomatology.

The objective of our study has been addressed to determine whether the major dimensions/traits of personality as defined by the alternative five factor (AFF) [18] model––Neuroticism-Anxiety, Activity, Sociability, Impulsive Sensation-Seeking, and Aggression-Hostility––would differentiate individuals with SCD from those of the general population (GP) using the Zuckerman-Kuhlman Personality Questionnaire (ZKPQ) [19].

The SCD sample was drawn from a cohort of participants [20] from the Fundació ACE Healthy Brain Initiative (FACEHBI), a longitudinal study comprising the assessment of biomarkers, risk factors, lifestyle, and cognition in individuals with SCD aged older than 49 years. This cohort has shown APOE *ε*4 (apolipoprotein with polymorphic alleles are the main genetic determinants of AD) enrichment, suggesting that a pool of potential AD patients could probably be nested in this sample [21].

To ensure that memory complaints were not due to affective symptomatology but to subjective perception of cognitive decline, participants scoring over the Spanish cut-off on the Hospital Anxiety and Depression Scale (HADS) [22] were not included in the study. The GP sample was part of a broader study designed to obtain the Spanish norms of the ZKPQ [23]. The present study deepens the knowledge on the characteristics of individuals with SCD and explores the relationship between personality and SCD.

## 2. Materials and Methods

### 2.1. Participants

Participants with SCD were recruited from the FACEHBI project [20], which consisted of 200 individuals. For the purpose of the present study, only those having completed the ZKPQ were included in the SCD group (*n* = 187). Comparing those who have responded the ZKPQ with those who have not responded it, both groups did not differ on the Mini-Mental State Examination (MMSE) [24] (Mann–Whitney *U* test, *p* = .241), age (Mann–Whitney *U* test, *p* = .655), and sex (Fisher exact test, *p* = .379).

SCD was defined as (a) age older than 49 years, (b) a score of ≥8 on the Spanish modified questionnaire of Memory Failures of Everyday (MFE-30) [25], (c) a score of ≥27 on the Spanish version of the MMSE, (d) a score of 0 on the Clinical Dementia Rating (CDR) Scale [26], (e) a performance within the average range expected for age and educational level on the Fundació ACE Neuropsychological Battery (NBACE) [27, 28], (f) were literate, and (g) had completed the self-administered questionnaire ZKPQ.

The exclusion criteria for the SCD group were (a) having a score of ≥11 in either of the two scales of the HADS [22], (b) having a history of alcoholism, epilepsy, or current serious medical or psychiatric illness, (c) displaying auditory or visual impairments interfering with neuropsychological assessment, and (d) evidencing functional impairment due to cognitive decline, with a score higher than 3 on the Blessed Dementia Rating Scale (BDRS) [29, 30]. Further description of inclusion and exclusion criteria is provided in Rodríguez-Gómez et al. [20].

Participants constituting the GP sample were extracted from a broader study of a total of 1,678 participants [23]. As this population was intended to obtain the Spanish norms, no inclusion/exclusion criteria were applied.

The final total sample consisted of 187 participants (71 men, 116 women) meeting criteria for SCD and 187 participants from the GP sample, matched by sex and age. The mean age of the total sample was 65.89 (SD = 7.29). With regard to the level of education of the SCD and GP samples, 21.9% and 40.1% completed elementary school, 27.8% and 20.9% high school, and 50.3% and 39% a bachelor's degree, respectively. In the present study, we did not exclude any participant due to the scores on the Infrequency scale and all were Caucasian.

### 2.2. Measures

#### 2.2.1. Psychological Assessments

Personality was assessed by the ZKPQ [23], a questionnaire designed to measure normal personality and not psychological abnormalities. The ZKPQ consists of 99 items in a “true-false” answering format. This questionnaire evaluates five major personality factors and includes an Infrequency scale that allows eliminating subjects with careless responding. This scale ensures that none of the responses is affected by set bias and it controls inaccurate responding. This Infrequency scale (10 items) detects inattention to the task and can be used as a validity measure for the individual test taker. The items in this scale are mostly exaggerated, true scored, socially desirable, but unlikely to be completely true statements about anyone. This scale is highly skewed, with most scores around 0 or 1.

The five scales can be described as follows: (1) Neuroticism-Anxiety (N-Anx, 19 items) items describe frequent emotional upset, tension, worry, fearfulness, indecision, lack of self-confidence, and sensitivity to criticism. (2) Activity (Act, 17 items) items describe the need for general activity, an inability to relax and do nothing when the opportunity arises, and a preference for hard and challenging work, an active, busy life, and a high energy level. (3) Sociability (Sy, 17 items) items describe outgoingness at parties, the number of friends one has and the amount of time spent with them, and a preference for being with others as opposed to being alone and engaging in solitary activities. (4) Impulsive Sensation-Seeking (ImpSS, 19 items) items involve a lack of planning and the tendency to act impulsively without thinking and the seeking for excitement, novel experiences, and the willingness to take risks for these types of experiences. These items are expressed in a general manner and do not describe specific activities such as drinking or sex. (5) Aggression-Hostility (Agg-Host, 17 items) items describe a readiness to express verbal aggression; rude, thoughtless, or antisocial behavior; vengefulness and spitefulness; having a quick temper and impatience towards others. This test has shown good psychometric properties in Spanish samples, with internal consistency alpha coefficients ranging from 0.70 to 0.85 [31] and high consensual validity parameters [32]. The factorial structure has also been replicated in Spanish samples with congruence coefficients to USA samples, ranging from 0.84 to 0.96 [23].

#### 2.2.2. Blood Sampling, APOE Genotyping

As detailed elsewhere [21], in the SCD sample, blood samples after fasting have been obtained in all the visits for standard biochemical analysis, determination of blood amyloid species, APOE genotyping, and DNA banking.

Genomic DNA was extracted from 200 *μ*l of human whole blood using Maxwell® 16 Blood DNA purification kit (Promega) according to the manufacturer's instructions. APOE rs7412 and rs42358 markers were genotyped using real-time PCR. PCR reactions were performed in a final volume of 5 *μ*l, using 11 ng of genomic DNA, 0.3 *μ*M of each amplification primer, and 2.65 *μ*l of 2X SYBR Fast Master Mix (Kapa Biosystems). We used an initial denaturation step at 95°C for 2 min, followed by 33 cycles at 95°C for 10 s, and at 69°C for 30 s. Melting curves were 95°C for 15 s (ramping rate 5.5°Cs), 45°C for 15 s (ramping rate of 5.5°Cs−1), and 95°C for 15 s (ramping rate of 5.5°Cs−1). In the last step of each melting curve, a continuous fluorimetric register was performed by the system at one acquisition register per each degree Celsius. Melting peaks and genotype calls were obtained using the Eco Real-Time PCR system (Illumina).

### 2.3. Procedure

Data from participants with SCD were obtained from the FACEHBI project. Participants were referred by their general health practitioner to Fundació ACE in order to be assessed for possible cognitive impairment, or approached Fundació ACE through an Open House Initiative in which any citizen can sign up for free cognitive screening without the need of medical referral. This initiative encompasses a community service and a recruitment strategy for research studies. A neuropsychologist administered the NBACE, HADS, MFE-30, and the ZKPQ, and a neurologist administered the MMSE and the neurological exploration. A nurse performed blood extraction for amyloid. Both procedures were performed at Fundació ACE. The SCD participants gave written consent and research protocol was approved by the ethics committee of the Hospital Clinic i Provincial (Barcelona, Spain) (EudraCT: 2014-000798-38).

Data from the GP sample were obtained from a broader study designed to obtain the Spanish norms of the ZKPQ [23]. This sample consisted of a total 1,678 participants, 741 males (44.2%) and 937 females (55.8%). From the GP sample, 187 participants were randomly extracted and matched by sex and age to participants of FACEHBI study.

Participants for the GP sample were contacted from very different sites (classroom, home, while waiting for a yearly health check, leisure associations, etc.) and asked to answer the ZKPQ questionnaire. The questionnaire was provided with written instructions and an introductory letter explaining globally the goal of the study (“the study you will collaborate in attempts to evaluate the functioning of an American questionnaire in our culture”). Most of the questionnaires were administered in a group situation and others individually. In this latter situation, the subject also received a prepaid envelope, which had to be posted.

Participation in the FACEHBI and the GP group was both voluntary and without remuneration. Questionnaires of the SCD group were signed and keep confidential. Questionnaires of the GP group were obtained anonymously.

### 2.4. Statistical Analysis

To assess differences between SCD and GP groups on the ZKPQ factors, we performed a two-tailed independent Student's *t*-test and calculated Cohen's *d*. In a second step, we executed a logistic regression analysis and as predictors we introduced the personality variables that previously obtained a significant effect in the bivariate analysis. Educational level was incorporated in the model (as dummy variable) because it was heterogeneously distributed in both groups. As both samples were matched by age and sex, these two variables were not considered as covariates. A conditional entrance strategy was applied to select the final significant personality model. The dichotomous status of participants (coded as 1 = SCD, 0 = GP) was the dependent factor. We used the SPSS v20, and the alpha risk assumed was 5%.

## 3. Results

Table 1 shows means, standard deviations, and alphas for the SCD and GP groups. Group comparisons showed differences between groups on the Neuroticism-Anxiety and Activity scales, with the SCD group scoring significantly lower on both scales.

Table 2 shows correlations between ZKPQ scales for both groups. While several statistically significant correlations were found in the GP group, in the SCD group, only Agg-Host with N-Anx and ImpSS showed a statistically significant and positive association (.38 and .14, respectively).

With regard to the SCD group, comparing APOE *ε*4 carriers with noncarriers, results indicated that they did not differ in any of the ZKPQ personality variables (for all comparisons *p* > .23).

To determine the specific contribution of Neuroticism-Anxiety and Activity scales to discriminate between SCD and GP groups, we conducted a logistic regression analysis. Since both groups differed in educational level (*χ*^2^ = 14.62, *p* = .001), we included this variable in the multivariate analysis as an adjustment factor. The educational level was classified into three categories; hence, we created two dummy variables that were introduced into the model. The Activity scale was the only variable that remained statistically significant in the final model (Wald = 5.50, *p* = .019, OR = 1.08, 95%CI = 1.01‐1.15).

## 4. Discussion

The SCD has been established as a risk factor for developing AD and it has been identified as a potential early stage of the disease [1]. To our knowledge, this is the first study that has analyzed the relationship between SCD and personality assessed by the ZKPQ by comparing individuals referring SCD with a general population sample matched by sex and age.

The results of the present study revealed that participants with SCD scored lower on the Neuroticism-Anxiety and Activity scales than the GP sample. Furthermore, when we determined the specific contribution of these two scales in discriminating between both groups, only the Activity scale remained in the model, indicating that individuals with SCD refer a low energy level and a dislike for an active and busy life.

Our results indicate that those participants referring SCD have lower levels of general activity, including doing things when the opportunity arises, a dislike for hard and challenging work, for an active and busy life, and display a low energy level. A longitudinal study would help to disentangle the question whether this low physical activity is due to personality characteristics *per se* or, in contrast, it is an incipient preclinical sign of AD [33], as lower activity can be consistent with changes intrinsic to the disease process itself [34]. Overall, it becomes difficult to determine which complaints underlie AD, because there is a close relationship between SCD complaints and other variables such as personality traits [35].

Consequently, the cross-sectional design of our study is a limitation and further longitudinal studies are needed to determine whether individuals with SCD and scoring low on the activity personality trait may have an increased risk for developing future cognitive decline and AD.

However, the strengths of the present study must also be mentioned. First, all participants underwent an extensive neuropsychological protocol to ensure that they had a neuropsychological preserved performance, and subjective cognitive complaints assessed through a standardized questionnaire. Second, each participant from the SCD group was matched by age and sex to a participant from the GP. Third, the major dimensions of personality were assessed within the alternative five factor model in which the scale level analyses of the ZKPQ [19, 36] allowed to examine which specific trait of the personality dimensions was most strongly related to AD.

Studies on lifestyle risk factors for developing AD have shown that decreased general activity is an important risk factor as they are low educational level, diabetes, smoking, midlife obesity, hypertension, and depression [10]. Furthermore, the recent WHO guidelines for prevention of dementia states that physical activity should be recommended to adults with normal cognition in order to reduce the risk of cognitive decline. It is important to take into account the potential risk factors, which would help in the prevention of dementia, as well as interventions that delay the cognitive decline [37].

The results of the present study suggest that personality traits might help to characterize individuals with SCD; hence, including the assessment of personality in SCD protocols would be welcomed. Studies provide support for that physical activity [38] and cognitive activities [39] offer some reduction in AD. Clinicians would prescribe modifying therapies, such as personalized physical exercise [40] or cognitive stimulation, to delay the onset of cognitive impairment or dementia. Early care programs might introduce actions to encourage people to increase their cognitive and physical activities.

## 5. Conclusions

The present study showed that compared to the general population, Activity trait is lower in individuals with SCD, indicating lower preference for an active and busy life, and lower energy level. Personality test may be helpful in characterizing persons with SCD. The assessment of personality may result in an SCD *plus* feature, which may serve as an upgrading strategy for future research.

## Figures and Tables

**Table 1:** Comparison of the ZKPQ scale scores between the SCD and control groups, Cronbach's alphas, and effect sizes.

ZKPQ scale	SCD group (*n* = 187)		*α*	GP group (*n* = 187)		*α*	*t*	*p*	Cohen's *d*
	Mean	SD		Mean	SD				
N-Anx	7.05	4.40	.84	8.06	4.68	.83	2.41	.033^∗^	.22
Act	7.67	3.50	.74	8.56	3.25	.76	2.55	.011^∗^	.26
Sy	6.68	3.57	.76	6.84	3.29	.76	.45	.652	.05
ImpSS	6.46	4.09	.82	6.97	3.70	.87	1.25	.214	.13
Agg-Host	6.20	3.20	.74	6.30	3.13	.77	.33	.744	.03
Inf	2.85	2.20	—	3.09	1.95	—	1.09	.275	.11

Note: SCD: subjective cognitive decline; GP: general population; N-Anx: Neuroticism-Anxiety; Act: Activity; Sy: Sociability; ImpSS: Impulsivity Sensation-Seeking; Agg-Host: Aggression-Hostility; Inf: Infrequency. ^∗^*p* < .05.

**Table 2:** Correlations between ZKPQ scales by group.

ZKPQ	N-Anx	Act	Sy	ImpSS	Agg-Host
N-Anx		-.02	-.02	.07	.38^∗∗^
Act	.09		.08	-.06	.06
Sy	-.06	.13		-.05	.05
ImpSS	.19^∗∗^	.32^∗∗^	.25^∗∗^		.14^∗^
Agg-Host	.28^∗∗^	.24^∗∗^	.06	.29^∗∗^	

Note: up-diagonal correlations in the FACEHBI group. Below-diagonal correlations in the GP group. N-Anx: Neuroticism-Anxiety; Act: Activity; Sy: Sociability; ImpSS: Impulsivity Sensation-Seeking; Agg-Host: Aggression-Hostility. ^∗^*p* < .05; ^∗∗^*p* < .005.

## Data Availability

Previously reported participant data were used to support this study and are available with the DOI: 10.14283/jpad.2016.122 and 10.1037/t06537-000. These prior studies (and datasets) are cited at relevant places within the text as references [20, 23].

## References

[1] Jessen F., Amariglio R. E., van Boxtel M. (2014). A conceptual framework for research on subjective cognitive decline in preclinical Alzheimer's disease. *Alzheimer’s & Dementia*.

[2] Mitchell A. J., Beaumont H., Ferguson D., Yadegarfar M., Stubbs B. (2014). Risk of dementia and mild cognitive impairment in older people with subjective memory complaints: meta-analysis. *Acta Psychiatrica Scandinavica*.

[3] Geerlings M. I., Jonker C., Bouter L. M., Ader H. J., Schmand B. (1999). Association between memory complaints and incident Alzheimer’s disease in elderly people with normal baseline cognition. *American Journal of Psychiatry*.

[4] Jonker C., Launer L. J., Hooijer C., Lindeboom J. (1996). Memory complaints and memory impairment in older individuals. *Journal of the American Geriatrics Society*.

[5] Reid L., Maclullich A. M. (2006). Subjective memory complaints and cognitive impairment in older people. *Dementia and Geriatric Cognitive Disorders*.

[6] Kim J. M., Stewart R., Kim S. W., Yang S. J., Shin I. S., Yoon J. S. (2006). A prospective study of changes in subjective memory complaints and onset of dementia in South Korea. *American Journal of Geriatric Psychiatry*.

[7] Mark R. E., Sitskoorn M. M. (2013). Are subjective cognitive complaints relevant in preclinical Alzheimer's disease? A review and guidelines for healthcare professionals. *Reviews in Clinical Gerontology*.

[8] Schmand B., Jonker C., Geerlings M. I., Lindeboom J. (1997). Subjective memory complaints in the elderly: depressive symptoms and future dementia. *British Journal of Psychiatry*.

[9] Zufferey V., Donati A., Popp J. (2017). Neuroticism, depression, and anxiety traits exacerbate the state of cognitive impairment and hippocampal vulnerability to Alzheimer’s disease. *Alzheimer's & Dementia: Diagnosis, Assessment & Disease Monitoring*.

[10] Terracciano A., Sutin A. R., An Y. (2014). Personality and risk of Alzheimer's disease: New data and meta-analysis. *Alzheimer's & Dementia*.

[11] Snitz B. E., Weissfeld L. A., Cohen A. D. (2015). Subjective cognitive complaints, personality and brain amyloid-beta in cognitively normal older adults. *American Journal of Geriatric Psychiatry*.

[12] Hollands S., Lim Y. Y., Buckley R. (2015). Amyloid-*β* related memory decline is not associated with subjective or informant rated cognitive impairment in healthy adults. *Journal of Alzheimer’s Disease*.

[13] Perrotin A., Mormino E. C., Madison C. M., Hayenga A. O., Jagust W. J. (2012). Subjective cognition and amyloid deposition imaging: a Pittsburgh compound B positron emission tomography study in normal elderly individuals. *Archives of Neurology*.

[14] Amariglio R. E., Becker J. A., Carmasin J. (2012). Subjective cognitive complaints and amyloid burden in cognitively normal older individuals. *Neuropsychologia*.

[15] Alegret M., Rodríguez O., Espinosa A. (2015). Concordance between subjective and objective memory impairment in volunteer subjects. *Journal of Alzheimer’s Disease*.

[16] Duberstein P. R., Chapman B. P., Tindle H. A. (2011). Personality and risk for Alzheimer’s disease in adults 72 years of age and older: a 6-year follow-up. *Psychology and Aging*.

[17] Caselli R. J., Langlais B. T., Dueck A. C. (2018). Personality changes during the transition from cognitive health to mild cognitive impairment. *Journal of the American Geriatrics Society*.

[18] Zuckerman M., Kuhlman D. M., Joireman J., Teta P., Kraft M. (1993). A comparison of three structural models for personality: the big three, the big five, and the alternative five. *Journal of Personality and Social Psychology*.

[19] Gomà-i-Freixanet M., Valero S., Puntí J., Zuckerman M. (2004). Psychometric properties of the Zuckerman–Kuhlman Personality Questionnaire in a Spanish sample. *European Journal of Psychological Assessment*.

[20] Rodríguez-Gómez O., Sanabria A., Pérez-Cordón A. (2017). FACEHBI: a prospective study of risk factors, biomarkers and cognition in a cohort of individuals with subjective cognitive decline. Study rationale and research protocols. *The Journal of Prevention of Alzheimer’s Disease*.

[21] Moreno-Grau S., Rodríguez-Gómez O., Sanabria A. (2017). Exploring APOE genotype effects on AD risk and b-amyloid burden in individuals with subjective cognitive decline: the FACEHBI study baseline results. *Alzheimer’s & Dementia*.

[22] De Las Cuevas Castresana C., García-Estrada Pérez A., Gonzáles de Rivera J. L. (1995). “Hospital anxiety and depression scale” y Psicopatología Afectiva. *Anales de Psiquiatría*.

[23] Gomà-i-Freixanet M., Valero S. (2008). Spanish normative data of the Zuckerman–Kuhlman Personality Questionnaire in a general population sample. *Psicothema*.

[24] Blesa R., Pujol M., Aguilar M. (2001). Clinical validity of the 'mini-mental state' for Spanish speaking communities. *Neuropsychologia*.

[25] Lozoya-Delgado P., Ruiz-Sánchez de León J. M., Pedrero-Pérez E. J. (2012). Validation of a cognitive complaints questionnaire for young adults: the relation between subjective memory complaints, prefrontal symptoms and perceived stress. *Revista de Neurologia*.

[26] Morris J. C. (1993). The clinical dementia rating (CDR): current version and scoring rules. *Neurology*.

[27] Alegret M., Espinosa A., Vinyes-Junqué G. (2012). Normative data of a brief neuropsychological battery for Spanish individuals older than 49. *Journal of Clinical and Experimental Neuropsychology*.

[28] Alegret M., Espinosa A., Valero S. (2013). Cut-off scores of a brief neuropsychological battery (NBACE) for Spanish individual adults older than 44 years old. *PLoS One*.

[29] Brugnolo A., Morbelli S., Arnaldi D. (2014). Metabolic correlates of Rey auditory verbal learning test in elderly subjects with memory complaints. *Journal of Alzheimer’s Disease*.

[30] Blessed G., Tomlinson B. E., Roth M. (1968). The association between quantitative measures of dementia and of senile change in the cerebral grey matter of elderly subjects. *British Journal of Psychiatry*.

[31] Gomà-i-Freixanet M., Valero S., Muro A., Albiol S. (2008). Zuckerman-Kuhlman Personality Questionnaire: psychometric properties in a sample of the general population. *Psychological Reports*.

[32] Gomà-i-Freixanet M., Wismeijer A., Valero S. (2005). Consensual validity parameters of the Zuckerman–Kuhlman Personality Questionnaire: evidence from self-reports and spouse reports. *Journal of Personality Assessment*.

[33] Valecha N., Olives J., Tort A. (2015). Informants’ perception of subjective cognitive decline helps to discriminate preclinical Alzheimer’s disease from normal aging. *Journal of Alzheimer’s Disease*.

[34] Kulmala J., Solomon A., Kareholt I. (2014). Association between mid- to late life physical fitness and dementia: evidence from the CAIDE study. *Journal of Internal Medicine*.

[35] Barnes D. E., Yaffe K. (2011). The projected effect of risk factor reduction on Alzheimer’s disease prevalence. *The Lancet Neurology*.

[36] Martínez Ortega Y., Gomà-i-Freixanet M., Valero S. (2016). Psychometric properties and normative data of the Zuckerman–Kuhlman Personality Questionnaire in a psychiatric outpatient sample. *Journal of Personality Assessment*.

[37] World Health Organization and Alzheimer’s Disease International (2012). *Dementia: a public health priority*.

[38] Sofi F., Valecchi D., Bacci D. (2011). Physical activity and risk of cognitive decline: a meta-analysis of prospective studies. *Journal of Internal Medicine*.

[39] Sajeev G., Weuve J., Jackson J. W. (2016). Late-life cognitive activity and dementia: a systematic review and bias analysis. *Epidemiology*.

[40] Sobol N. A., Dall C. H., Høgh P. (2018). Change in fitness and the relation to change in cognition and neuropsychiatric symptoms after aerobic exercise in patients with mild Alzheimer’s disease. *Journal of Alzheimer’s Disease*.

